# Association between COVID-19 outbreak and preterm birth rates in a low-infection cohort from a tertiary Shanghai maternity center: a retrospective study

**DOI:** 10.3389/fped.2026.1770233

**Published:** 2026-08-26

**Authors:** Rui-Hong Xue, Jue Ma, Juan Li, Lin Zhang

**Affiliations:** 1 International Peace Maternity and Child Health Hospital, School of Medicine, Shanghai Jiao Tong University, Shanghai, China; 2 Shanghai Key Laboratory of Embryo Original Diseases, Shanghai, China; 3 School of Medicine, Institute of Birth Defects and Rare Diseases, Shanghai Jiao Tong University, Shanghai, China

**Keywords:** COVID-19, pandemic, pregnancy, preterm birth, preterm birth rate

## Abstract

**Objective:**

To evaluate the association between the COVID-19 pandemic and preterm birth (PTB) rates, stratified by gestational age, in a low-infection pregnant population delivering at a non-designated COVID-19 maternity center.

**Design:**

A hospital-based retrospective cohort study was conducted, comparing deliveries during the pandemic period (January 2020–April 2022) with the pre-pandemic period (January 2017–April 2019). Logistic regression models were used to assess crude and adjusted associations, with singleton-only sensitivity analyses performed to address confounding by multiple gestation.

**Results:**

A total of 67,238 deliveries were included, comprising 29,310 pandemic and 37,928 pre-pandemic births. Overall PTB rates were similar between the two periods (7.5% vs. 7.4%; adjusted odds ratio [AOR] = 1.0, 95% confidence interval [CI]: 1.0–1.1, *P* = 0.66). Preterm birth excluding extremely preterm birth (EPTB) (28^+0^–36⁺^6^ weeks) rates also remained stable (7.3% vs. 7.3%; AOR = 1.0, 95% CI: 0.9–1.1, *P* = 0.95). In contrast, the rate of EPTB (22^+0^–27⁺^6^ weeks) was significantly higher during the pandemic (0.2% vs. 0.1%), with a nearly threefold increased risk after adjustment (AOR = 2.8, 95%CI: 1.7–4.7, *P* < 0.001). This association persisted in the singleton-only cohort (AOR = 3.2, 95% CI: 1.8–5.9, *P* < 0.001), despite the rarity of this outcome.

**Conclusions:**

Among this low-infection cohort reflecting pandemic measures rather than viral effects, overall and preterm birth excluding EPTB rates remained stable during the pandemic, though a significant increase in extremely preterm birth (<28 weeks) risk was noted, indicating a subtype-specific effect requiring further validation.

## Introduction

In December 2019, an unexplained pneumonia confirmed as coronavirus disease 2019 (COVID-19) broke out in Wuhan City in Hubei Province (1). In Shanghai, a city-wide lockdown was declared on January 24, 2020 and ended on March 24, 2020 (2). Though the city lockdown in Shanghai effectively controlled the spread of virus in the following more than two years, a new viral genome BA.2, a sub-lineage of the omicron variant of SARS-CoV-2 (B.1.1.159), appeared to be more transmissible and swept this city mainly from March 2022 on (3), and strict lockdown was reinstated at the end of March 2022 (4). According to the Shanghai Municipal Health Commission, as of May 4, 2022, 593,336 cases have been identified, including 538,450 asymptomatic carriers. A total of 503 deaths were attributed to COVID-19 (5). Compared with the early stages of the COVID-19 outbreak, Shanghai imposed far more stringent lockdown measures commencing in late March 2022, requiring residents to stay indoors during this containment period (4).

Pregnant women are a uniquely vulnerable population, and COVID-19 infection may increase their risks of adverse maternal and fetal outcomes; meanwhile, the pandemic exacerbated psychological distress among expectant mothers (6, 7). After the outbreak of COVID-19, the cesarean section rates slightly increased in a non-infected population compared with the prior year (8).

Preterm birth (PTB) is a significant cause of infant mortality and morbidity (9). Globally, one in ten babies was born preterm, and the preterm birth rate has been worldwide increasing (10). China was reported to have the second highest number of preterm births, more than 1 million babies born preterm per year (11). The preterm birth rate had been increasing by 1.1% per year from 1990 to 2016 (12). However, whether the risk of preterm birth is increased among low-infection pregnant women during the COVID-19 pandemic (or associated lockdown measures) remains controversial (13–18). This study aimed to evaluate whether these indirect COVID-19-related factors were associated with alterations in the epidemiology of preterm birth (PTB) in a low-infection population.

## Method

### Data collection

A hospital-based retrospective study was conducted to investigate the association between the COVID-19 outbreak and preterm birth (PTB) rates. After obtaining approval from the local ethics committee on human research, we used data from the digital birth registration system at International Peace Maternity and Child Health Hospital, School of Medicine, Shanghai Jiao Tong University—a large tertiary maternity center in Shanghai, China. The hospital caters to both low- and high-risk pregnant women, with approximately 10,000–15,000 deliveries annually. Post-delivery, maternal characteristics and neonatal information are routinely documented in the digital birth registration system. Notably, it is not a medical records system: patient diagnoses are incomplete. However, maternal and neonatal delivery-related data are relatively complete and reliable. As one of Shanghai's five prenatal diagnosis centers, our institution is not a designated facility for managing COVID-19-positive pregnant women. Preterm birth rates across different regions of Shanghai have ranged from 5% to 10% in recent years.

The data available to us comprised women who delivered at the center between 1 January 2017 and 30 April 2022. To avoid potential differences in preterm birth risk across calendar months, as well as possible seasonal effects on the incidence of preterm birth (19), data from May 2019 to December 2019 were not included, thereby ensuring that the calendar months included in the pre-pandemic and pandemic period study periods were fully aligned. Enrolled patients were stratified into two groups based on the COVID-19 outbreak: the pandemic group (deliveries between January 2020 and April 2022) and the pre-pandemic group (deliveries in the corresponding pre-outbreak period: January 2017 to April 2019). For records with missing or extreme gestational age values, we retrieved and verified information through the inpatient medical record system using hospitalization numbers, followed by data imputation and correction, to minimize missing data and diagnostic inaccuracies attributable to manual entry errors.

The primary exposure of interest was the COVID-19 pandemic period, defined as deliveries occurring between January 2020 and April 2022. This exposure represents a composite real-world construct encompassing multiple concurrent pandemic-related factors: municipal public health lockdown policies, hospital-level reorganization of obstetric care services, reduced maternal mobility and changes in daily lifestyle, and pandemic-related psychosocial stress. Given our institution was not a designated facility for managing COVID-19-infected pregnant women during the study period, we performed a thorough secondary review of electronic health records to identify cases of maternal COVID-19 infection at delivery. Due to limitations in diagnostic coding within the digital birth registration system, definitive identification of all potential COVID-19 cases was not feasible. To our knowledge, merely 2 cases of confirmed maternal COVID-19 delivered emergently at our center during our study period, yielding an extremely low infection prevalence in our cohort. We judged that the potential impact of direct COVID-19 infection on preterm birth outcomes was negligible.

All deliveries recorded in the hospital's routine digital birth registration system during the study period (January 2017–April 2022) were initially included. During data cleaning, we performed a secondary review of delivery records (including neonatal count and birth data) to identify and exclude cases of multiple gestation, a well-established strong risk factor for preterm birth. A total of 1,840 multiple gestation deliveries were excluded, resulting in a singleton-only cohort of 65,398 deliveries. This step was taken to eliminate confounding by multiple gestation, as part of our prespecified sensitivity analysis plan.

### Outcome measures

Preterm birth (PTB) was defined as delivery prior to 37 weeks, including extremely preterm birth (EPTB) (22^+0^ to 27⁺^6^ weeks' gestation) and preterm birth excluding EPTB (28^+0^ to 36⁺^6^ weeks' gestation). All preterm birth including spontaneous labor, preterm premature rupture of the membranes (PPROM), and iatrogenic preterm delivery (labor induction or cesarean section for maternal/fetal indications) (20). In the present study, PTB was classified as delivery prior to 37 weeks' gestation, including both vaginal and cesarean deliveries. PTB rates were calculated for each group and compared statistically.

Women with confirmed COVID-19 were diagnosed via nucleic acid testing, with samples processed by the hospital's on-site COVID-19 laboratory and results available within 6–8 h. As our institution was not a designated facility for managing COVID-19-infected pregnant women, only a limited number of known pregnant women with confirmed COVID-19 delivered emergently during the entire study period. Accordingly, the low-infection population was operationally defined as pregnant women who delivered at our maternity center.

### Data analysis

Sample size considerations: Assuming a pre-pandemic EPTB rate of 0.06%, a minimum clinically detectable odds ratio of 2.5 (two-sided *α* = 0.05, power = 0.80), the required sample size was calculated using the method of Fleiss for binary outcomes. The calculation indicated that approximately 58,000 deliveries would be needed to detect such an effect. Multivariable adjustments were made for maternal age, parity, and residential status to mitigate potential confounding effects. The proportion of missing data for all variables was <5% for parity, and residential status. Missing values were sporadic and unrelated to the study exposure (pandemic period) or outcome (preterm birth), and were either missing completely at random (MCAR) or missing at random (MAR). Multiple logistic regression was used to assess the association between the COVID-19 pandemic (pandemic vs. pre-pandemic period) and the risk of preterm birth. We assessed multicollinearity and model fit; no violations were detected. First, unadjusted odds ratios (ORs) were calculated to explore the crude association between the pandemic and preterm birth. Second, adjusted ORs (AORs) with 95% confidence intervals (CIs) were estimated to account for potential confounding by the maternal age, parity, and residential status. Based on a directed acyclic graph (DAG; Supplementary Figure S1), mode of delivery was identified as a mediator on the causal pathway between pandemic-related exposures and preterm birth, and was therefore not included as a covariate in the primary model to avoid over-adjustment. Furthermore, subgroup analyses were performed to explore the stability of the primary finding across key populations. The Wald test was used to determine statistical significance for variables associated with preterm birth risk, while the *Z*-score test was employed to compare cesarean section rates between the two periods. The differences in preterm birth rates between the lockdown group and each control year were compared using the Pearson's chi-squared test or Fisher's exact test, where appropriate (expected cell frequency <5).

To assess the robustness of the primary findings, several sensitivity analyses were conducted, including subgroups and singleton-only analysis. For each sensitivity analysis, logistic regression models were used to estimate the odds ratio (OR) and 95% confidence interval (CI) for the association between the pandemic period and preterm birth, adjusting for the same covariates as the primary model.

A *P*-value <0.05 was considered statistically significant. All multivariable logistic regression analyses were performed using the Statistical Package for Social Sciences (SPSS) version 23.0 (IBM, USA).

## Results

### Study population and data collection

A hospital-based retrospective analysis was performed using data from the digital birth registration system. A total of 67,399 deliveries occurred at the maternity center during the study period. A total of 21 cases with missing or erroneous gestational age data were verified and corrected by cross-referencing the inpatient medical record system. After excluding cases with missing/extreme parity (*n* = 142), and missing/undetermined residential status (*n* = 19), the final analysis included 67,238 deliveries. Among these, 29,310 deliveries occurred during the pandemic period (January 2020–April 2022), and 37,928 deliveries occurred during the pre-pandemic period (January 2017–April 2019). A limited number of known pregnant women with confirmed COVID-19 delivered emergently at our hospital in April 2022, as our institution was not a designated facility for managing COVID-19-infected pregnant women, and all such deliveries were conducted in isolated delivery rooms. All study data were retrieved from the hospital's digital birth registration system, which recorded only 2 COVID-19-positive pregnant women throughout the research period, both admitted during the strict April 2022 lockdown. While confirmed COVID-19 patients received rigorous isolation management, incomplete case capture via this registration system may lead to recording bias and underestimation of actual infection numbers.

### Stratified preterm birth rates by maternal and clinical factors

The overall preterm birth rate across the study population was 7.4% (4,978/67,238). Significantly higher preterm birth rates were observed in several subgroups compared with their respective reference groups (all *P* < 0.05). Women aged ≥35 years had a higher PBR (9.0%) than those aged <35 years (6.9%). Cesarean section (CS) deliveries were associated with a substantially higher PBR (10.2%) compared with vaginal birth (VB) (5.0%). Immigrant women had a higher PBR (8.2%) than permanent residents (7.2%). Women with parity ≥1 also had a slightly higher PBR (7.9%) than primiparous women (parity = 1, 7.2%) (Table 1).

**Table 1:** Stratified preterm birth rates (PBR) according to maternal clinical characteristics among 67,238 deliveries.

Total	Characteristics	Classification	Women, *n*, (%)	Preterm, *n*	PBR^ϯ^,%	Overall PBR,%
Total^†^ *N* = 67,238	Age	˂35 (reference)	51,061 (75.94)	3,528	6.9%	7.4% (4,978/67,238)
		≥35	16,177 (24.06)*	1,450	9.0%	
	Parity	1 (reference)	46,582 (69.28)	3,357	7.2%	
		≥1	20,656 (30.72)	1,621	7.9%	
	Mode	VB^§^ (reference)	36,014 (53.56)	1,786	5.0%	
		CS^ɸ^	31,224 (46.44)*	3,192	10.2%	
	Residential status	Residents (reference)	51,091 (75.99)	3,655	7.2%	
		Immigrants	16,147 (24.01)*	1,323	8.2%	

ɸ CS indicates cesarean delivery; ^§^VB indicates vaginal delivery; ^ϯ^PBR indicates preterm birth rates.

* *P* < 0.05 (compared with reference during every characteristic unit).

† This sum does not necessarily equal the sample size for all variables because some data may be missing.

### Cesarean section rates pre- and during the pandemic

The CS rate was 48.0% in the pandemic period (January 2020–April 2022) vs. 45.2% in the pre-pandemic period (January 2017–April 2019), representing a statistically significant increase following the pandemic outbreak (*P* < 0.001) (Table 2).

**Table 2:** Cesarean section rates before and during the pandemic.

Total	Group	Modes	Deliveries, *n*, (%)	CS rates,%	*P* value
Total^†^ *N* = 67,238	Pandemic	CS^ɸ^	14,069 (48.0)	48.0%	<0.001
	*N* = 29,310	VB^§^	15,241 (52.0)		
	Pre-pandemic	CS^ɸ^	17,155 (45.23)	45.2%	
	*N* = 37,928	VB^§^	20,773 (54.77)		

ɸ CS indicates cesarean section; ^§^VB indicates vaginal birth.

† This sum does not necessarily equal the sample size for all variables because some data were missing.

### Preterm birth rates: pre-pandemic vs. pandemic periods

A total of 29,310 women gave birth during the pandemic (PBR = 7.5%) and 37,928 during the pre-pandemic period (PBR = 7.4%). Multiple logistic regression showed an odds ratio (95% CI) of 1.0 (1.0, 1.1) and an adjusted odds ratio (95% CI) of 1.0 (1.0, 1.1), with corresponding *P*-values of 0.70 and 0.66, indicating the COVID-19 outbreak did not significantly alter preterm birth risk after adjustment for maternal age, parity, and birthplace (Table 3).

**Table 3:** Preterm birth rates (PBR) before and during the pandemic (unadjusted for mode of delivery).

Group	Modes	Births, *n*, (%)	Preterm, *n*, (%)	PBR^ϯ^,%	Odds ratio (95% CI)	*P*	Adjusted odds ratio (95% CI)*	*P*
Pandemic *N* = 29,310	CS^ɸ^	14,069 (48.0)	1,410 (10.0)	7.5%	1.0 (1.0, 1.1)	0.7	1.0 (1.0, 1.1)	0.7
	VB^§^	15,241 (52.0)	773 (5.1)					
Pre-pandemic *N* = 37,928	CS^ɸ^	17,155 (45.2)	1,782 (10.4)	7.4%				
	VB^§^	20,773 (54.8)	1,013 (4.9)					

ɸ CS indicates cesarean delivery; ^§^VB indicates vaginal delivery; ^ϯ^PBR indicates preterm birth rates.

* AOR was adjusted for maternal age, parity, birthplace.

### Preterm birth rate comparison: April 2022 lockdown vs. control period

We performed a separate analysis comparing preterm birth rates (PBR) during the strict April 2022 lockdown with April data from 2017 to 2021 as controls. No significant differences were found for overall or cesarean-specific PBR across all pairwise comparisons (all *P* > 0.05). The overall PBR stood at 7.4% in April 2022, vs. a range of 7.0%–8.2% in control Aprils. Cesarean-related PBR was 9.8% during lockdown, compared with 10.0%–11.4% in prior years. These results indicate the April 2022 lockdown exerted no meaningful effect on preterm birth risk (Supplementary Table S1).

### Preterm birth rates among cesarean deliveries: pre vs. pandemic

The CS preterm rate was 10.4% (1,782/17,155) pre-pandemic and 10.0% (1,410/14,069) during the pandemic, with no statistically significant difference observed (*P* = 0.29). This suggests the COVID-19 pandemic did not alter preterm risk for cesarean deliveries (Supplementary Table S2).

To further assess the stability of the primary findings, a series of sensitivity analyses and subgroup analyses stratified by maternal age, residential status, and parity were conducted (Supplementary Tables S3–S9).

### Sensitivity analysis adjusted for mode of delivery

The overall PBR was 7.5% (2,183/29,310) during the pandemic period and 7.4% (2,795/37,928) during the pre-pandemic period. The adjusted odds ratio (AOR) for preterm birth, adjusted for maternal age, parity, birthplace, and mode of delivery was 1.0 (95% CI: 0.9–1.1, *P* = 0.75), indicating no significant difference in preterm birth risk between the two periods (Supplementary Table S3).

### Subgroup analysis by maternal age

Among women aged <35 years (Supplementary Table S4), the PBR was 7.0% (1,575/22,597) during the pandemic period and 6.9% (1,953/28,464) during the pre-pandemic period. No significant association between the pandemic and preterm birth risk was observed (unadjusted OR = 1.0, 95% CI: 1.0–1.1, *P* = 0.63). Among women aged ≥35 years (Supplementary Table S5), the PBR was 9.1% (608/6,713) in the pandemic period and 8.9% (842/9,464) in the pre-pandemic period, with no significant difference detected (unadjusted OR = 1.0, 95% CI: 0.9–1.1, *P* = 0.90).

### Subgroup analysis by residential status

Among permanent residents (Supplementary Table S6), the PBR was 7.0% (1,520/21,842) during the pandemic period and 7.3% (2,135/29,249) during the pre-pandemic period, showing no significant association (unadjusted OR = 1.0, 95% CI: 0.9–1.0, *P* = 0.19). Among immigrant women (Supplementary Table S7), the crude unadjusted analysis demonstrated a statistically significant association between the pandemic period and preterm delivery (OR = 1.2, 95% CI: 1.1–1.3, *P* = 0.003). After adjustment for maternal age and parity, the adjusted odds ratio remained significant with a consistent effect magnitude (AOR = 1.2, 95% CI: 1.1–1.3, *P* = 0.004). Stratified by study period, the preterm birth rate reached 8.9% (663 preterm cases out of 7,468 total births) during the pandemic, compared with 7.6% (660 preterm cases out of 8,679 total births) in the pre-pandemic cohort. Collectively, these findings illustrate that the elevated preterm birth risk among immigrant women during the pandemic persisted independently after accounting for maternal age and parity.

### Subgroup analysis by mode of delivery

In the vaginal birth subgroup (Supplementary Table S8), the PBR was 5.1% (773/15,241) during the pandemic period and 4.9% (1,013/20,773) during the pre-pandemic period, with no significant difference observed (unadjusted OR = 1.0, 95% CI: 1.0–1.2, *P* = 0.40). In the cesarean delivery subgroup (Supplementary Table S9), the PBR was 10.0% (1,410/14,069) in the pandemic period and 10.4% (1,782/17,155) in the pre-pandemic period. No significant association between the pandemic and preterm birth risk was found (unadjusted OR = 1.0, 95% CI: 0.9–1.0, *P* = 0.39), confirming that the increased cesarean section rate during the pandemic did not result in a higher preterm birth risk. Overall, all sensitivity and subgroup analyses supported the primary conclusion that the COVID-19 pandemic was not significantly associated with an increased risk of preterm birth.

### Sensitivity analysis including full 2019 calendar year

A sensitivity analysis was conducted to assess the robustness of the primary findings by incorporating 10,881 additional deliveries from May to December 2019 into the pre-pandemic group, expanding the pre-pandemic period to January 2017–December 2019 while keeping the pandemic period (January 2020–April 2022) unchanged. The preterm birth rate (PBR) was 7.5% (2,183/29,310) in the pandemic group and 7.2% (3,507/48,809) in the expanded pre-pandemic group. Logistic regression analysis showed no significant association between the pandemic period and preterm birth risk (OR = 1.0, 95% CI: 1.0–1.1, *P* = 0.17). These results confirm that the exclusion of May–December 2019 in the primary analysis did not introduce bias, and the null association between the COVID-19 pandemic and preterm birth rates remains fully robust when the full study period is considered (Supplementary Table S10).

### Sensitivity analysis excluding multiple gestations

A sensitivity analysis was conducted to evaluate the robustness of our primary findings by excluding multiple gestations, a well-established strong risk factor for preterm birth (PTB). A total of 1,840 cases of multiple gestation were excluded from the original cohort, leaving 65,398 singleton pregnancies for analysis. The preterm birth rate (PBR) was 6.1% (1,735/28,631) in the pandemic group and 5.7% (2,105/36,767) in the pre-pandemic group. Logistic regression analysis showed no statistically significant increase in preterm birth risk associated with the pandemic period among singleton pregnancies (OR = 1.1, 95% CI: 1.0–1.1, *P* = 0.07). These results are consistent with the primary analysis, confirming that the null association between the COVID-19 pandemic and preterm birth rates remains robust in singleton pregnancies (Supplementary Table S11).

### Extremely preterm birth rates: pre-pandemic vs. pandemic

Of the total cohort of 67,238 deliveries (29,310 during the pandemic period and 37,928 in the pre-pandemic period), the extremely preterm birth rate was significantly higher during the pandemic period than before the pandemic: 0.2% (46/29,310) vs. 0.1% (21/37,928). Logistic regression analysis showed that the pandemic period was associated with a nearly threefold increased risk of extremely preterm birth. In the unadjusted model, the odds ratio (OR) was 2.8 (95% CI: 1.7–4.8, *P* < 0.001). This association remained highly significant after adjusting for maternal age, parity, and residential status (birthplace) (adjusted OR = 2.8, 95% CI: 1.7–4.7, *P* < 0.001) (Table 4).

**Table 4:** Extremely preterm birth (<28 week) rates before and during the pandemic.

Total	Group	Extremely Preterm, *n*	EPTB^※^,%	OR	*P*	AOR^＃^	*P*
Total *N* = 67,238	Pandemic	46	0.2	2.8 (1.7, 4.8)	<0.001	2.8 (1.7, 4.7)	<0.001
	*N* = 29,310						
	Pre-pandemic	21	0.1				
	*N* = 37,928						

※ EPTB indicates extremely preterm birth (<28 week) rates.

＃ AOR was adjusted for maternal age, parity, and birthplace.

### Extremely preterm birth rates after excluding multiple gestations

After exclusion of multiple gestation cases, the overall singleton cohort included 65,398 deliveries: 28,631 births in the pandemic period and 36,767 births in the pre-pandemic period. The extremely preterm birth rate was markedly elevated during the pandemic, standing at 0.1% (38 out of 28,631) compared with only 0.0% (15 out of 36,767) before the pandemic. Logistic regression revealed a robust positive association between the pandemic period and higher extremely preterm birth risk. The crude unadjusted odds ratio (OR) was 3.3 (95% CI: 1.8–5.9, *P* < 0.001). After adjustment for maternal age, parity, and birthplace, the adjusted odds ratio (AOR) remained 3.2 (95% CI: 1.8–5.9, *P* < 0.001) with persistent statistical significance. Collectively, these findings confirm that the elevated risk of extremely preterm delivery during the pandemic cannot be attributed to multiple gestations, and the independent association holds after accounting for core maternal covariates (Supplementary Table S12).

### Preterm birth excluding EPTB rates: pre-pandemic vs. pandemic

The overall study population contained 67,238 deliveries: 29,310 births during the pandemic and 37,928 births before the pandemic. The rate of non-extremely preterm birth was almost indistinguishable across the two timeframes, standing at 7.3% (2,137 out of 29,310) in the pandemic cohort and 7.3% (2,774 out of 37,928) in the pre-pandemic cohort. Logistic regression failed to identify any statistically significant correlation between the pandemic period and risk of preterm delivery excluding EPTB. The crude unadjusted odds ratio (OR) was 1.0 (95% CI: 0.9–1.1, *P* = 0.91). After adjustment for maternal age, parity, and birthplace, the adjusted odds ratio (AOR) remained 1.0 (95% CI: 0.9–1.1, *P* = 0.95), still without statistical significance. Collectively, these data demonstrate that the COVID-19 pandemic was not associated with altered risk of preterm birth excluding EPTB (28^+0^ to 36^+6^ weeks), showing no elevation in preterm risk within this gestational subgroup (Supplementary Table S13).

### Subgroup analyses of extremely preterm birth risk

The overall EPTB rate increased from 0.1% (21/37,928) pre-pandemic to 0.2% (46/29,310) during the pandemic (AOR = 2.8, 95% CI: 1.7–4.7, *P* < 0.001). Stratified analyses revealed prominent subgroup heterogeneity. Significantly higher pandemic EPTB risk was found in local women (AOR = 4.3, *P* < 0.001), advanced maternal age (AMA, AOR = 4.0, *P* = 0.003) and non-AMA mothers (AOR = 2.3, *P* = 0.011), primiparous (AOR = 2.5, *P* = 0.002) and multiparous women (AOR = 3.9, *P* = 0.020), as well as women with non-cesarean delivery (AOR = 8.3, *P* < 0.001). In contrast, no significant pandemic effect was observed among non-local women (AOR = 0.7, *P* = 0.536) and those undergoing cesarean delivery (AOR = 1.0, *P* = 0.957). All subgroups were non-mutually exclusive (Supplementary Table S14).

### Stratified analyses of very and moderate-to-late preterm birth

We provided stratified risk estimates for two preterm subtypes (28⁺^0^–31⁺^6^ weeks very preterm birth [VPB] and 32⁺^0^–36⁺^6^ weeks moderate-to-late preterm birth [MLPTB]) across pre-pandemic (reference, *N* = 37,928) and pandemic (*N* = 29,310) periods, with a total cohort of 67,238 deliveries. The VPB rate slightly declined from 0.8% pre-pandemic to 0.7% during the pandemic, with an adjusted odds ratio (AOR) of 0.9 (95% CI: 0.8–1.1, *P* = 0.25). The MLPTB marginally rose from 6.6% to 6.6%, yielding an AOR of 1.0 (95% CI: 1.0–1.1, *P* = 0.74). All AORs were adjusted for maternal age, parity, and birthplace. Neither VPB nor MLPTB showed a statistically significant pandemic-associated risk shift, indicating the COVID-19 pandemic exerted no independent effect on the risk of non-extremely preterm delivery (Supplementary Table S15).

### Stratified EPTB risks across pandemic subphases

The pre-pandemic reference group (*N* = 37,928) had an EPTB rate of 0.1%. Pandemic Phase 1 (2020, *N* = 13,467, rate = 0.1%) showed significantly higher EPTB risk (AOR = 2.4, 95%CI 1.3–4.5, *P* = 0.006). Pandemic Phase 2 (2021, *N* = 12,288, rate = 0.2%) carried the strongest significant elevation (AOR = 3.5, 95%CI 2.0–6.4, *P* < 0.001). For Pandemic Phase 3 (Jan–Mar 2022, *N* = 2,673, rate = 0.2%), the elevated risk was marginally non-significant (AOR = 2.7, 95%CI 0.9–7.9, *P* = 0.068). Notably, no EPTB cases were recorded during the strict April 2022 lockdown (*N* = 882), with no corresponding adjusted risk estimate available (Supplementary Table S16).

### Sensitivity analysis excluding COVID-19-positive pregnant women

After exclusion of COVID-19-positive pregnant women, the EPTB rate was still markedly higher in the pandemic cohort (0.2%) than the pre-pandemic group (0.1%), with a significant elevated adjusted risk (AOR = 2.8, 95% CI: 1.7–4.7, *P* < 0.001). By contrast, overall preterm birth rates were similar across groups (7.5% vs. 7.4%, AOR = 1.0, *P* = 0.67). Consistent with our main results, the sensitivity test verified that the pandemic only raised the risk of extremely preterm birth, without altering overall preterm birth risk (Supplementary Table S17).

## Discussion

In this retrospective study, we found that the overall preterm birth rate (<37 weeks) and preterm birth excluding EPTB rate (28–36⁺^6^ weeks) remained unchanged during the COVID-19 pandemic in the low-infection population. Notably, the risk of extremely preterm birth <28 weeks was significantly elevated amid the pandemic. Our findings highlight divergent subtype-specific impacts of the COVID-19 outbreak on preterm birth, underscoring the necessity of stratifying preterm birth by severity rather than treating it as a homogeneous entity, as well as sustained attention to pandemic-related adverse perinatal outcomes in the global context.

Previous data reported that pregnant women who contract COVID-19 were at an increased risk of pregnancy complications and mortality. For women with severe course of SARS-CoV-2 infection in pregnancy, risk of cesarean section and hospital and intensive care unit admission increased (21). Allotey et al. (22) reported a spontaneous preterm birth rate of 6% in women with COVID-19. In a large population-based study, COVID-19 diagnosis increased the risk of very preterm births, preterm births, and early term birth, particularly among people with medical comorbidities, and appeal for vaccination to be prioritized for birthing persons (23). Published data suggested that symptomatic pregnant patients with COVID-19 were at increased risk of more severe illness compared with nonpregnant women (24, 25). SARS-CoV-2, once bound to the ACE2 receptor, downregulates the expression of ACE2 receptor which leads to increased risk of vascular, endothelial, and microcirculatory dysfunction including vasoconstriction, which could further increase risk of miscarriages, premature births and intrauterine growth restrictions (26). Symptomatic COVID-19 should be considered a risk factor for preterm birth, possibly due to an increase in iatrogenic deliveries for maternal indications (27). With only two confirmed maternal COVID-19 cases detected in our cohort (both from April 2022) and retained in analysis due to data diagnostic limitations, direct viral infection exerted negligible influence on preterm birth rates, although we acknowledge constraints in our dataset may restrict complete case capture.

The lockdown during the pandemic has also brought multiple adverse effects on pregnant women, both physically and mentally. During the lockdown period there was a 43.2% reduction in hospital admissions when compared with the control period and a 66.4% reduction in referred obstetric emergencies compared with the same calendar period in the previous year (28). Existing studies report a marked rise in anxiety and depression prevalence across the general population (29, 30). A growing body of evidence further links the COVID-19 pandemic and lockdown restrictions to worsened mental health among pregnant women, such as heightened psychological distress and depressive symptoms (7, 31).

However, the global evidence about preterm birth rates during the COVID-19 pandemic compared with the pre-pandemic period remains contradictory and urgently needs clarification (16, 17, 32). A population-based prospective study found unchanged overall preterm birth rates during the pandemic (15), consistent with our observations of preterm birth excluding EPTB rates in this large low-infection Shanghai maternity cohort. A Shanghai cohort study found elevated preterm risks among uninfected second-trimester pregnant women under lockdown (14). Another report also noted higher rates of term premature rupture of membranes (PROM) in uninfected women delivering during the 2022 lockdown vs. the same timeframe in 2021 (33). Reduced preterm birth rates during the pandemic are unlikely to stem from delayed care-seeking; they may instead result from less maternal physical activity and fewer medically induced preterm deliveries (34). Dong et al. (35) reported that COVID-19 lockdown was associated with a slightly shorter gestational age and a moderately increased risk of preterm birth; however, that study did not perform separate subgroup analyses for extremely preterm birth (EPTB, <28 weeks' gestation), which restricted in-depth investigation of pandemic-related effects on extremely preterm birth. A Dutch study found reduced medically indicated very and extremely preterm singleton births during the 2020 lockdown, alongside more spontaneous extremely preterm deliveries (<28 weeks) in multiple pregnancies (36). A significantly lower rate of extremely premature deliveries during the lockdown compared with the prior mean rate in the previous years was also reported (16).

Discrepancies between our findings and previous Shanghai studies reporting elevated preterm birth (PTB) risk during lockdown may be attributed to differences in study design, separate statistical analyses by distinct gestational age diagnostic criteria for PTB, study population, and observation timeframe. Most prior regional studies focused exclusively on the strict lockdown period (March–May 2022) and only assessed overall PTB, whereas our study covered the full pandemic period (January 2020–April 2022) and performed separate statistical analyses based on different gestational age diagnostic thresholds for PTB (extremely preterm birth <28 weeks and preterm birth excluding EPTB 28^+0^–36⁺^6^ weeks). Notably, we confirmed no significant increase in overall PTB or preterm birth excluding EPTB even during the strict lockdown month of April 2022, and the elevated risk of extremely preterm birth observed in our cohort was not concentrated in this strict lockdown phase. Our cohort represented a low-infection population from a specialized tertiary maternity center with an extremely low prevalence of maternal COVID-19 infection, which differs from the more heterogeneous populations in other studies that may have included a higher proportion of COVID-19-infected pregnant individuals and more unmeasured confounders. Additionally, our large sample size provided adequate precision for effect estimation, whereas small sample sizes in previous studies may have led to imprecise estimates and increased random error. Furthermore, our comprehensive adjustment for confounding factors, separate analyses by PTB gestational age diagnostic criteria, and multiple robustness checks (including singleton-only analysis and lockdown-specific analysis) further enhanced the reliability of our findings compared with prior investigations.

The contrasting association trends observed for extremely preterm birth (EPTB) and very preterm birth (VPB) are unlikely to represent true biological disparities. The apparent protective tendency within the VPB subgroup is largely attributable to case ascertainment bias, selective survival effects and insufficient statistical power, hence excessive interpretation of this subgroup result is inappropriate, and the analysis centers on the stable and credible association linked to EPTB. When delivery mode is redefined as a mediating factor instead of a confounding variable and excluded from core analytical models, the correlation relevant to EPTB remains steady, whereas the statistical association for non-extremely preterm birth disappears entirely. This distinction supports the proposed causal pathway: delivery mode exerts a mediating effect on preterm birth excluding EPTB that are heavily shaped by obstetric clinical decisions, while EPTB stems chiefly from severe intrinsic pathological conditions independent of delivery routes. Intensified antenatal monitoring, variable clinical intervention standards and inconsistent case detection may artificially strengthen the risk signal of EPTB, and residual confounding together with survival bias contributes to the weak negative association seen in VPB populations. The relatively small number of EPTB cases limits statistical capacity and prohibits exhaustive covariate adjustment. To avoid model overfitting, only core recognised confounders are incorporated into analyses following standard statistical principles. Even under this sample constraint, the primary EPTB association maintains statistical validity, and additional singleton-only analyses further confirm the finding is not driven by multiple gestation. Analyses stratified by research period are regarded only as exploratory observations rather than confirmatory evidence, as stratification further reduces sample availability. This methodological limitation is clearly acknowledged, and validation via larger multicenter cohorts is recommended for future research. Altogether, the heterogeneous findings across gestational age cutoffs highlight the necessity of categorising preterm birth into standard gestational subgroups rather than pooling all non-extremely preterm events, which enables a more rational and comprehensive interpretation of differential risks across preterm subtypes.

Geng et al. (37) reported that pandemic lockdowns prolonged home waiting time in women with term PROM, which was associated with elevated maternal C-reactive protein. Similarly, lockdown-induced delays in care-seeking for second-trimester vaginal infections or preterm PROM may raise infection-related extremely preterm birth risk, warranting further research. Notably, some studies linked second-trimester lockdown exposure to higher preterm birth risk (14). However, among the 67 extremely preterm birth cases included in the analysis, 21 occurred during the pre-pandemic period and 46 during the pandemic period, no cases occurred during April 2022 (the strict lockdown period), suggesting strict lockdown might not be the direct cause. Other pandemic-related factors may underlie the elevated risk of extremely preterm birth, which merits further investigation.

The study has several notable strengths. First, this large hospital-based retrospective cohort enrolled over 67,000 deliveries with a month-matched study design, a homogeneous low-infection pregnant population, and standardized obstetric care provision. These features greatly enhanced statistical power, improved result robustness, and minimized confounding from seasonal variations and selection bias. The extended observation period covered the full pandemic timeline, supporting a reliable and comprehensive assessment of pandemic-related changes in preterm birth. Second, we performed separate analyses for extremely preterm birth and preterm birth excluding EPTB based on distinct gestational age diagnostic thresholds, which helped identify subtype-specific effects that would have been overlooked by only evaluating overall preterm birth. Third, comprehensive confounder adjustment (for maternal age, parity, residential status, and mode of delivery) and multiple rigorous sensitivity analyses—including singleton-only and strict lockdown-specific analyses—were applied to validate result stability, further reducing potential bias.

However, several notable limitations of this study should be acknowledged. First, the main limitation is the single-center, retrospective design, which restricts generalizability. Second, although we conducted separate statistical analyses for extremely preterm birth and preterm birth excluding EPTB based on standard gestational age diagnostic thresholds, we were unable to differentiate spontaneous from iatrogenic preterm births owing to the lack of standardized subtype coding in the routine birth registration system. These two preterm birth subtypes have distinct etiological mechanisms and may respond differently to pandemic-related factors such as lockdown measures, health care reorganization, and psychosocial stress, which limits the mechanistic interpretation of our findings. Third, the study relied on a routine digital birth registration system that only records basic delivery-related information, and we lacked comprehensive maternal clinical data. Socioeconomic status, maternal stress, and healthcare access during the pandemic were also not available in our dataset. Key preterm birth risk factors, including pre-existing and gestational comorbidities, assisted reproductive technology conception status, maternal mental health, prenatal care adherence, and COVID-19 vaccination history, could not be reliably collected. In addition, diagnostic coding constraints limited the complete identification of all maternal COVID-19 cases, although the infection prevalence in our cohort was extremely low. These data deficiencies may lead to residual confounding and restrict the evaluation of indirect pandemic-related impacts. Fourth, although we excluded multiple gestation cases and performed a singleton-only sensitivity analysis to verify the robustness of our results, the absence of several key unmeasured confounders in the primary model may still introduce residual confounding, and a type II statistical error for weak pandemic-associated effects cannot be ruled out. Fifth, the role of cesarean section in the causal pathway of medically indicated preterm birth remains conceptually debatable. We performed a sensitivity analysis by excluding delivery mode from the regression model, yet residual confounding related to clinical decision-making for preterm cesarean delivery could not be fully eliminated. Sixth, this study had a small number of EPTB events (67 cases) and a wide 95% CI (1.7–4.7), so results need verification in larger samples. Seventh, formal high-risk pregnancy referral records were unavailable in our hospital database, meaning we lack direct quantitative data to account for potential shifts in high-risk case inflow across study periods. As our center was not designated for COVID-19-positive parturients, pandemic triage policies, mobility restrictions, and altered health care accessibility may have changed the referral ratio of low-risk and high-risk pregnant women. Potential selection bias should be considered when interpreting and generalizing the findings. Future multi-center prospective studies based on complete electronic medical records are warranted to collect detailed data on maternal comorbidities, conception modes, COVID-19 infection status, and classified preterm birth subtypes, so as to further validate our results and clarify the true impact of the COVID-19 pandemic on preterm birth risk.

In conclusion, in this low-infection cohort of pregnant women, overall and preterm birth excluding EPTB rates remained stable during the pandemic, though a significant increase in extremely preterm birth (<28 weeks) risk was noted, indicating a subtype-specific effect requiring further validation.

Table 1 presents preterm birth rates (PBR) stratified by key clinical and maternal characteristics among the 67,238 deliveries included in the final analysis. Of these women, 24.1% were aged ≥35 years, 69.3% were primiparous (parity = 1), 46.4% delivered by cesarean section, and 76.0% were permanent residents. The overall preterm birth rate during the study period was 7.4% (4,978/67,238).

Table 2 presents cesarean section (CS) rates during and before the COVID-19 pandemic. The CS rate was 48.0% in the pandemic period, compared with 45.2% in the pre-pandemic period. A significant increase in CS rates was observed following the COVID-19 outbreak (*Z*-score test, *Z* = 7.1, *P* < 0.001).

Table 3 presents preterm birth rates (PBR) during the pandemic (January 2020–April 2022) and the pre-pandemic period (January 2017–April 2019). A total of 29,310 women gave birth during the pandemic (PBR = 7.5%) and 37,928 during the pre-pandemic period (PBR = 7.4%). Multiple logistic regression showed an odds ratio (95% CI) of 1.0 (1.0, 1.1) and an adjusted odds ratio (95% CI) of 1.0 (1.0, 1.1), with corresponding *P*-values of 0.70 and 0.66, indicating the COVID-19 outbreak did not significantly alter preterm birth risk after adjustment for maternal age, parity, and birthplace.

Table 4 presents the extremely preterm birth (EPTB, defined as delivery at <28 weeks' gestation) rates before and during the COVID-19 pandemic. Of the total cohort of 67,238 deliveries (29,310 during the pandemic period and 37,928 in the pre-pandemic period), the extremely preterm birth rate was significantly higher during the pandemic period than before the pandemic: 0.2% (46/29,310) vs. 0.1% (21/37,928). Logistic regression analysis showed that the pandemic period was associated with a nearly threefold increased risk of extremely preterm birth. In the unadjusted model, the odds ratio (OR) was 2.8 (95% CI: 1.7–4.8, *P* < 0.001). This association remained highly significant after adjusting for maternal age, parity, and residential status (birthplace) (adjusted OR = 2.8, 95% CI: 1.7–4.7, *P* < 0.001).

## Data Availability

The original contributions presented in the study are included in the article/Supplementary Material, further inquiries can be directed to the corresponding author/s.
