# Supplementary material for: Association between COVID-19 outbreak and preterm birth rates in a low-infection cohort from a tertiary Shanghai maternity center: a retrospective study
Source: Front Pediatr. 2026 Aug 26;14:1770233. doi: 10.3389/fped.2026.1770233 (PMC13553850; doi:10.3389/fped.2026.1770233)
Supplement: Supplementary file 1 [file Supplementaryfile1.docx]

**Supplementary tables**

| Table S1 Preterm Birth Rate (PTB) and Preterm Birth Rate in Cesarean Deliveries Between April Lockdown Group and Control Group | | | | | | | | | | |
| --- | --- | --- | --- | --- | --- | --- | --- | --- | --- | --- |
| Groups | Month | Births, n | Preterm, n | PBR^ϯ^,% | P |  | CS^ɸ^ | Preterm | PBR^ϯ^,% | P |
| Lockdown | 2022.4 | 882 | 65 | 7.4 | >0.05 |  | 399 | 39 | 9.8 | >0.05 |
| Unlockdown | 2021.4 | 964 | 79 | 8.2 |  |  | 485 | 53 | 10.9 |  |
|  | 2020.4 | 1081 | 80 | 7.4 |  |  | 519 | 52 | 10.0 |  |
|  | 2019.4 | 1307 | 104 | 8.0 |  |  | 586 | 60 | 10.2 |  |
|  | 2018.4 | 1273 | 98 | 7.7 |  |  | 622 | 71 | 11.4 |  |
|  | 2017.4 | 1264 | 89 | 7.0 |  |  | 559 | 58 | 10.4 |  |
| ^ɸ^ ***CS indicates cesarean delivery;*** ^ϯ^***PBR indicates preterm birth rates.*** | | | | | | | | | | |
| Groups \| Overal PBR \| P value \| CS PBR \| P value; 2022.4 vs 2021.4 \| 7.4% vs 8.2% \| 0.5089 \| 9.8% vs 10.9% \| 0.5763; 2022.4 vs 2020.4 \| 7.4% vs 7.4% \| 0.9792 \| 9.8% vs 10.0% \| 0.9021; 2022.4 vs 2019.4 \| 7.4% vs 8.0% \| 0.6135 \| 9.8% vs 10.2% \| 0.8119; 2022.4 vs 2018.4 \| 7.4% vs 7.7% \| 0.7766 \| 9.8% vs 11.4% \| 0.4095; 2022.4 vs 2017.4 \| 7.4% vs 7.0% \| 0.7718 \| 9.8% vs 10.4% \| 0.7610 | | | | | | | | | | |

An additional, dedicated analysis was conducted focusing on April 2022 (a month under strict COVID-19-related lockdown), to investigate the differences in preterm birth rates (PBR) between the lockdown period and the control group. We detected no statistically significant differences in the overall preterm birth rate (PBR) or cesarean delivery (CS)-specific PBR when comparing the COVID-19 lockdown month (April 2022: overall PBR 7.4%, CS PBR 9.8%) separately to each non-lockdown April from 2017 to 2021. Specifically, the overall PBR of the control years ranged from 7.0% (April 2017) to 8.2% (April 2021), and the CS-specific PBR ranged from 10.0% (April 2020) to 11.4% (April 2018). All pairwise comparisons yielded P > 0.05, demonstrating that the April 2022 COVID-19 lockdown had no measurable impact on preterm birth rates in this cohort.

| Table S2 Comparison of Preterm Birth Rate (PTB) in Cesarean Deliveries Before and After the COVID-19 Pandemic | | | | | | | | |
| --- | --- | --- | --- | --- | --- | --- | --- | --- |
| Group | Births, n | Modes | n | % |  | Preterm, n | PBR^ϯ^,% | P |
| Pandemic | 29310 | CS^ɸ^ | 14069 | 48.0% |  | 1410 | 10.0% | P=0.29 |
| Pre-pandemic | 37928 | CS^ɸ^ | 17155 | 45.2% |  | 1782 | 10.4% |  |
| ^ɸ^ ***CS indicates cesarean delivery;*** ^ϯ^***PBR indicates preterm birth rates.*** | | | | | | | | |

Table S2 presents an additional analysis of preterm birth rates (PBR) specifically among cesarean section (CS) deliveries before and during the COVID-19 pandemic. During the pre-pandemic period, the preterm birth rate among CS deliveries was 10.4% (1782/17155); during the pandemic period, the preterm birth rate among CS deliveries was 10.0% (1410/14069). Statistical comparison showed no significant difference between the two periods (P = 0.29), indicating that the preterm birth rate among cesarean deliveries did not change significantly after the outbreak of the COVID-19 pandemic.

| Table S3 Sensitivity analysis of preterm birth rates before and after the COVID-19 pandemic with adjusted for mode of delivery | | | | | |
| --- | --- | --- | --- | --- | --- |
| Groups | Births, n | Preterm, n | PBR^ϯ^,% | Adjusted odds ratio (95% CI)* | P |
|  |  |  |  |  |  |
| Pandemic | 29310 | 2183 | 7.5% | 1.0(0.9, 1.1) | 0.75 |
| Pre-pandemic | 37928 | 2795 | 7.4% |  |  |
| ^ɸ^ CS indicates cesarean delivery; ^§^VB indicates vaginal delivery; ^ϯ^PBR indicates preterm birth rates. | | | | | |
| *AOR was adjusted for maternal age, parity, and birthplace and mode of delivery. | | | | | |

Table S3 presents the sensitivity analysis of preterm birth rates (PBR) between the pandemic and pre-pandemic periods, with adjustment for mode of delivery. The overall PBR was 7.5% (2183/29310) during the pandemic period and 7.4% (2795/37928) during the pre-pandemic period. The adjusted odds ratio (AOR) for preterm birth, adjusted for maternal age, parity, and birthplace, and mode of delivery was 1.0 (95% CI: 0.9–1.1, P=0.75), indicating no significant difference in preterm birth risk between the two periods.

| Table S4 Preterm birth rates in subgroup of maternal age < 35 before and during the pandemic | | | | |  |
| --- | --- | --- | --- | --- | --- |
|  | Group | Preterm, n | PBR^ϯ^,% | OR | P value |
| Total^†^ | Pandemic | 1575 | 7.0 | 1.0 （1.0， 1.1） | 0.63 |
|  | N=22597 |  |  |  |  |
|  |  |  |  |  |  |
| N=51061 | Pre-pandemic | 1953 | 6.9 |  |  |
|  | N=28464 |  |  |  |  |
|  |  |  |  |  |  |
| ^ϯ^PBR indicates preterm birth rates. | | | | | |

Table S4 presents preterm birth rates (PBR) in the subgroup of maternal age < 35 years. During the pandemic period (n=22,597), the PBR was 7.0% (1575/22597), compared with 6.9% (1953/28464) in the pre-pandemic period (n=28,464). Univariate analysis showed no statistically significant association between the pandemic and preterm birth risk in this subgroup (OR=1.0, 95% CI: 1.0–1.1, P=0.63). These results indicate that the COVID-19 pandemic did not increase the risk of preterm birth among younger pregnant women (< 35 years old).

| Table S5 Preterm birth rates in subgroup of maternal age ≥ 35 before and during the pandemic | | | | |  |
| --- | --- | --- | --- | --- | --- |
|  | Group | Preterm, n | PBR^ϯ^,% | OR | P |
| Total  N=16177 | Pandemic | 608 | 9.1 | 1.0 (0.9, 1.1) | 0.90 |
|  | N=6713 |  |  |  |  |
|  | Pre-pandemic | 842 | 8.9 |  |  |
|  | N=9464 |  |  |  |  |
| ^ϯ^PBR indicates preterm birth rates. | | | | | |

Table S5 displays PBR in the subgroup of maternal age ≥ 35 years. The PBR was 9.1% (608/6713) during the pandemic (n=6,713) and 8.9% (842/9464) in the pre-pandemic period (n=9,464). No significant difference in preterm birth risk was observed between the two periods (OR=1.0, 95% CI: 0.9–1.1, P=0.90). This suggests that elderly pregnant women (≥ 35 years old) did not face an elevated risk of preterm birth due to the pandemic.

| Table S6 Preterm birth rates in subgroup of residents before and during the pandemic | | | | |  |
| --- | --- | --- | --- | --- | --- |
|  | Group | Preterm, n | PBR^ϯ^,% | OR | P |
| Total  N=51091 | Pandemic | 1520 | 7.0 | 1.0 (0.9–1.0) | 0.19 |
|  | N=21842 |  |  |  |  |
|  | Pre-pandemic | 2135 | 7.3 |  |  |
|  | N=29249 |  |  |  |  |
| ^ϯ^PBR indicates preterm birth rates. | | | | | |

Table S6 summarizes PBR in the subgroup of permanent residents. During the pandemic (n=21,842), the PBR was 6.96% (1520/21842), slightly lower than the pre-pandemic rate of 7.3% (2135/29249) (n=29,249). However, the difference was not statistically significant (OR=1.0, 95% CI: 0.9–1.0, P=0.19). The OR value close to 1 and the 95% CI spanning 1 confirm that the pandemic did not significantly alter preterm birth risk among permanent resident pregnant women.

| Table S7 Preterm birth rates in subgroup of immigrants before and during the pandemic | | | | |  |  |  |
| --- | --- | --- | --- | --- | --- | --- | --- |
|  | Group | Preterm, n | PBR^ϯ^,% | OR | P | AOR^＃^ | P |
| Total  N=16147 | Pandemic | 663 | 8.9 | 1.2 (1.1, 1.3) | 0.003 | 1.2 (1.1, 1.3) | 0.004 |
|  | N=7468 |  |  |  |  |  |  |
|  | Pre-pandemic | 660 | 7.6 |  |  |  |  |
|  | N=8679 |  |  |  |  |  |  |
| ^ϯ^PBR indicates preterm birth rates.  ^＃^***AOR was adjusted for maternal age and parity.*** | | | | | | | |

Table S7 presents preterm birth rates (PBR) among immigrant pregnant women. The crude unadjusted analysis demonstrated a statistically significant association between the pandemic period and preterm delivery (OR=1.2, 95% CI: 1.1–1.3, P=0.003). After adjustment for maternal age and parity, the adjusted odds ratio remained significant with a consistent effect magnitude (AOR=1.2, 95% CI: 1.1–1.3, P=0.004). Stratified by study period, the preterm birth rate reached 8.9% (663 preterm cases out of 7,468 total births) during the pandemic, compared with 7.6% (660 preterm cases out of 8,679 total births) in the pre-pandemic cohort. Collectively, these findings illustrate that the elevated preterm birth risk among immigrant women during the pandemic persisted independently after accounting for maternal age and parity.

| Table S8 Preterm birth rates in subgroup of VB^§^ before and during the pandemic | | | | |  |
| --- | --- | --- | --- | --- | --- |
|  | Group | Preterm, n | PBR^ϯ^,% | OR | P |
| Total  N=36014 | Pandemic | 773 | 5.1 | 1.0 (1.0-1.2) | 0.40 |
|  | N=15241 |  |  |  |  |
|  | Pre-pandemic | 1013 | 4.9 |  |  |
|  | N=20773 |  |  |  |  |
| ^§^VB indicates vaginal delivery; ^ϯ^PBR indicates preterm birth rates. | | | | | |

Table S8 presents PBR in the subgroup of vaginal birth (VB). The PBR during the pandemic (n=15,241) was 5.1% (773/15241), compared with 4.9% (1013/20773) in the pre-pandemic period (n=20,773). No statistically significant difference was detected (OR=1.0, 95% CI: 1.0–1.2, P=0.40). These findings demonstrate that the pandemic did not increase the risk of preterm birth among women undergoing vaginal delivery.

| Table S9 Preterm birth rates in subgroup of CS^ɸ^ before and during the pandemic | | | | |  |
| --- | --- | --- | --- | --- | --- |
|  | Group | Preterm, n | PBR^ϯ^,% | OR | P |
| Total  N=31375 | Pandemic | 1410 | 10.0 | 1.0 (0.9, 1.0) | 0.39 |
|  | N=14069 |  |  |  |  |
|  | Pre-pandemic | 1782 | 10.4 |  |  |
|  | N=17155 |  |  |  |  |
| ^ϯ^PBR indicates preterm birth rates. | | | | | |

Table S9 displays PBR in the subgroup of cesarean delivery (CS). The PBR was 10.0% (1410/14069) during the pandemic (n=14,069) and 10.4% (1782/17155) in the pre-pandemic period (n=17,155). There was no significant association between the pandemic and preterm birth risk in this subgroup (OR=1.0, 95% CI: 0.9–1.0, P=0.39). This confirms that the increase in CS rate during the pandemic did not lead to a higher preterm birth risk among cesarean delivery recipients.

| Table S10 Sensitivity analysis of preterm birth rates with inclusion of the full 2019 calendar year (including May–December 2019) | | | | |  |
| --- | --- | --- | --- | --- | --- |
|  | Group | Preterm, n | PBR^ϯ^,% | OR | P |
| Total  N=78119 | Pandemic | 2183 | 7.5 | 1.0 (1.0, 1.1) | 0.17 |
|  | N=29310 |  |  |  |  |
|  | Pre-pandemic | 3507 | 7.2 |  |  |
|  | N=48809 |  |  |  |  |
| ^ϯ^PBR indicates preterm birth rates. | | | | | |

This sensitivity analysis was conducted to assess the robustness of the primary findings by incorporating 10,881 additional deliveries from May to December 2019 into the pre-pandemic group, expanding the pre-pandemic period to January 2017–December 2019 while keeping the pandemic period (January 2020–April 2022) unchanged. The preterm birth rate (PBR) was 7.5% (2183/29310) in the pandemic group and 7.2% (3507/48809) in the expanded pre-pandemic group. Logistic regression analysis showed no significant association between the pandemic period and preterm birth risk (OR = 1.0, 95% CI: 1.0–1.1, P = 0.17). These results confirm that the exclusion of May–December 2019 in the primary analysis did not introduce bias, and the null association between the COVID-19 pandemic and preterm birth rates remains fully robust when the full study period is considered.

| Table S11 Sensitivity analysis of preterm birth rates with exclusion of multiple gestation | | | | |  |
| --- | --- | --- | --- | --- | --- |
|  | Group | Preterm, n | PBR^ϯ^,% | OR | P |
| Total  N=65398 | Pandemic | 1735 | 6.1 | 1.1 (1.0, 1.1) | 0.07 |
|  | N=28631 |  |  |  |  |
|  | Pre-pandemic | 2105 | 5.7 |  |  |
|  | N=36767 |  |  |  |  |
| ^ϯ^PBR indicates preterm birth rates. | | | | | |

This sensitivity analysis was performed to evaluate the robustness of our primary findings by excluding multiple gestations—a well-documented, strong risk factor for preterm birth (PTB). A total of 1,840 multiple-gestation pregnancies were excluded from the original cohort, leaving 65,398 singleton pregnancies for analysis. The preterm birth rate (PBR) was 6.1% (1,735/28,631) in the pandemic group and 5.7% (2,105/36,767) in the pre-pandemic group. Logistic regression analysis revealed no statistically significant increase in PTB risk associated with the pandemic period among singleton pregnancies (OR = 1.1, 95% CI: 1.0–1.1, P = 0.07). These findings align with our primary analysis, confirming that the null association between the COVID-19 pandemic and overall preterm birth rates remains robust when the cohort is restricted to singleton pregnancies.

| Table S12 Extremely preterm birth (<28 week) rates before and during the pandemic with exclusion of multiple gestation | | | | | | | |
| --- | --- | --- | --- | --- | --- | --- | --- |
|  | Group | Extremely Preterm, n | EPBR^※^,% | OR | P | AOR^＃^ | P |
| Total  N=65398 | Pandemic | 38 | 0.1 | 3.3 (1.8, 5.9) | <0.001 | 3.2 (1.8, 5.9) | <0.001 |
|  | N=28631 |  |  |  |  |  |  |
|  | Pre-pandemic | 15 | 0.0 |  |  |  |  |
|  | N=36767 |  |  |  |  |  |  |
| ^ϯ^PBR indicates preterm birth rates; ^※^EPBR indicates extremely preterm birth (<28 week) rates.  ^＃^AOR ***was adjusted for maternal age, parity, and birthplace.*** | | | | | | | |

Table S12 presents extremely preterm birth (EPTB, delivery at <28 weeks’ gestation) rates before and during the COVID-19 pandemic among singleton pregnancies, with all multiple gestation cases excluded. The overall singleton cohort included 65,398 deliveries: 28,631 births in the pandemic period and 36,767 births in the pre-pandemic period. The extremely preterm birth rate was markedly elevated during the pandemic, standing at 0.1% (38 out of 28,631) compared with only 0.0% (15 out of 36,767) before the pandemic. Logistic regression revealed a robust positive association between the pandemic period and higher extremely preterm birth risk. The crude unadjusted odds ratio (OR) was 3.3 (95% CI: 1.8–5.9, P < 0.001). After adjustment for maternal age, parity, and birthplace, the adjusted odds ratio (AOR) remained 3.2 (95% CI: 1.8–5.9, P < 0.001) with persistent statistical significance. Collectively, these findings confirm that the elevated risk of extremely preterm delivery during the pandemic cannot be attributed to multiple gestations, and the independent association holds after accounting for core maternal covariates.

| Table S13 Preterm birth excluding EPTB (28-36^+6^ week) rates before and during the pandemic | | | | | | | |
| --- | --- | --- | --- | --- | --- | --- | --- |
|  | Group | Preterm birth excluding EPTB, n | PBR^※^,% | OR | P | AOR^＃^ | P |
| Total  N=67238 | Pandemic | 2137 | 7.3 | 1.0 (0.9, 1.1) | 0.91 | 1.0 (0.9, 1.1) | 0.95 |
|  | N=29310 |  |  |  |  |  |  |
|  | Pre-pandemic | 2774 | 7.3 |  |  |  |  |
|  | N=37928 |  |  |  |  |  |  |
| ^※^PBR indicates preterm birth excluding EPTB (28-36^+6^ week) rates.  ^＃^AOR ***was adjusted for maternal age, parity and birthplace.*** | | | | | | | |

Table S13 displays rates of preterm birth excluding extremely preterm birth (EPTB, gestational age 28^+0^ to 36^+6^ weeks) in the pre-pandemic and pandemic periods. The overall study population contained 67,238 deliveries: 29,310 births during the pandemic and 37,928 births before the pandemic. The rate of non-extremely preterm birth was almost indistinguishable across the two timeframes, standing at 7.3% (2,137 out of 29,310) in the pandemic cohort and 7.3% (2,774 out of 37,928) in the pre-pandemic cohort. Logistic regression failed to identify any statistically significant correlation between the pandemic period and risk of preterm delivery excluding EPTB. The crude unadjusted odds ratio (OR) was 1.0(95% CI: 0.9–1.1, P = 0.91). After adjustment for maternal age, parity, and birthplace, the adjusted odds ratio (AOR) remained 1.0 (95% CI: 0.9–1.1, P = 0.95), still without statistical significance. Collectively, these data demonstrate that the COVID-19 pandemic was not associated with altered risk of preterm birth excluding EPTB (28–36⁺⁶ weeks), showing no elevation in preterm risk within this gestational subgroup.

Table S14. Subgroup-specific risk estimates for extremely preterm birth (EPTB) (pre-pandemic vs. pandemic period)

| Subgroup | Pre-pandemic  (N=37928) | | Pandemic period (N=29310) | | Rate difference | OR | Adjusted OR (95% CI) | P value |
| --- | --- | --- | --- | --- | --- | --- | --- | --- |
|  | rate (%) | EPTB(n) | rate (%) | EPTB(n) |  |  |  |  |
| Overall | 0.1 | 21/37928 | 0.2 | 46/29310 | +0.1% | 2.8 | 2.8 (1.7–4.7) | <0.001 |
| Local women | 0.0 | 12/29249 | 0.2 | 39/21842 | +0.1% | 4.4 | 4.3 (2.2–8.2) | <0.001 |
| Non-local women | 0.1 | 9/8679 | 0.1 | 7/7468 | -0.0% | 0.9 | 0.7 (0.3–2.0) | 0.536 |
| Non-AMA | 0.1 | 15/28464 | 0.1 | 27/22597 | +0.1% | 2.3 | 2.3 (1.2–4.3) | 0.011 |
| AMA | 0.1 | 6/9464 | 0.3 | 19/6713 | +0.2% | 4.5 | 4.0 (1.6–10.1) | 0.003 |
| Primiparous women | 0.1 | 17/25609 | 0.2 | 35/20973 | +0.1% | 2.5 | 2.5 (1.4–4.5) | 0.002 |
| Multiparous women | 0.0 | 4/12319 | 0.1 | 11/8337 | +0.1% | 4.1 | 3.9 (1.3–12.4) | 0.020 |
| Non-cesarean delivery | 0.0 | 5/20773 | 0.2 | 31/15241 | +0.2% | 8.5 | 8.3 (3.2–21.4) | <0.001 |
| Cesarean delivery | 0.1 | 16/17155 | 0.1 | 15/14069 | +0.0% | 1.1 | 1.0 (0.5–2.1) | 0.957 |

Subgroups are not mutually exclusive; rates were calculated using each subgroup as the denominator. EPTB, extremely preterm birth; N, total number of deliveries in each subgroup; AMA, advanced maternal age.

| Table S15 Stratified analysis of preterm birth risks in very preterm birth (28^+0^–31^+6^ weeks) and moderate-to-late preterm birth (32^+0^–36^+6^ weeks) | | | | | | | |
| --- | --- | --- | --- | --- | --- | --- | --- |
|  | Group | ^※^VPB, % | AOR^＃^ | P | ^＆^MLPTB, % | AOR^＃^ | P |
| Total  N=67238 | Pandemic | 0.7% | 0.9 (0.8–1.1) | 0.25 | 6.6% | 1.0 (1.0–1.1) | 0.74 |
|  | N=29310 |  |  |  |  |  |  |
|  | Pre-pandemic  [Reference] | 0.8% |  |  | 6.6% |  |  |
|  | N=37928 |  |  |  |  |  |  |
| ^※^VPB indicates very preterm birth (28^+0^–31^+6^ weeks). ^＆^MLPTB indicates moderate-to-late preterm birth (32^+0^–36^+6^ weeks).  ^＃^AOR ***was adjusted for maternal age, parity and birthplace.*** | | | | | | | |

Table S15 provides stratified risk estimates for two preterm subtypes (28⁺⁰–31⁺⁶ weeks very preterm birth [VPB] and 32⁺⁰–36⁺⁶ weeks moderate-to-late preterm birth [MLPTB]) across pre-pandemic (reference, N=37,928) and pandemic (N=29,310) periods, with a total cohort of 67,238 deliveries. The VPB rate slightly declined from 0.8% pre-pandemic to 0.7% during the pandemic, with an adjusted odds ratio (AOR) of 0.9 (95% CI: 0.8–1.1, P=0.25). The MLPTB marginally rose from 6.6% to 6.6%, yielding an AOR of 1.0 (95% CI: 1.0–1.1, P=0.74). All AORs were adjusted for maternal age, parity, and birthplace. Neither VPB nor MLPTB showed a statistically significant pandemic-associated risk shift, indicating the COVID-19 pandemic exerted no independent effect on the risk of non-extremely preterm delivery.

| Table S16 Stratifies extremely preterm birth (EPTB) risks across the pre-pandemic control phase and four distinct pandemic sub-periods | | | | | |
| --- | --- | --- | --- | --- | --- |
| Phase | Deliveries | EPTB Cases | EPTB Rate (%) | AOR (95%CI) | P-value |
| Pre-pandemic (Control) | 37,928 | 21 | 0.1 | 1.0 (Reference) | - |
| Pandemic Phase 1 (year 2020) | 13467 | 18 | 0.1 | 2.4 (1.3–4.5) | 0.006 |
| Pandemic Phase 2 (year 2021) | 12288 | 24 | 0.2 | 3.5 (2.0–6.4) | <0.001 |
| Pandemic Phase 3 (2022.1-2022.3) | 2673 | 4 | 0.2 | 2.7 (0.9–7.9) | 0.068 |
| Pandemic Phase 3 (2022.4) | 882 | 0 | 0 | - | - |

Table S16 stratifies extremely preterm birth (EPTB) risks across the pre-pandemic control phase and four distinct pandemic sub-periods. The pre-pandemic reference group (N=37,928) had an EPTB rate of 0.1%. Pandemic Phase 1 (2020, N=13,467, rate=0.1%) showed significantly higher EPTB risk (AOR=2.4, 95%CI 1.3–4.5, P=0.006). Pandemic Phase 2 (2021, N=12,288, rate=0.2%) carried the strongest significant elevation (AOR=3.5, 95%CI 2.0–6.4, P<0.001). For Pandemic Phase 3 (Jan–Mar 2022, N=2,673, rate=0.2%), the elevated risk was marginally non-significant (AOR=2.7, 95%CI 0.9–7.9, P=0.068). Notably, no EPTB cases were recorded during the strict April 2022 lockdown (N=882), with no corresponding adjusted risk estimate available.

| Table S17 Sensitivity analysis of EPTB and preterm birth risks after exclusion of COVID-19-confirmed cases | | | | | | | |
| --- | --- | --- | --- | --- | --- | --- | --- |
|  | Group | ^※^EPTB, % | AOR^＃^ | P | ^＆^Preterm Birth, % | AOR^＃^ | P |
| Total  N=67236 | Pandemic | 0.2% | 2.8 (1.7 – 4.7) | <0.001 | 7.5% | 1.0 (1.0–1.1) | 0.67 |
|  | N=29308 |  |  |  |  |  |  |
|  | Pre-pandemic  [Reference] | 0.1% |  |  | 7.4% |  |  |
|  | N=37928 |  |  |  |  |  |  |
| ^※^EPTB indicates extremely preterm birth < 28 weeks (22^+0^ to 27⁺^6^ weeks’ gestation). ^＆^Preterm birth is defined as delivery at 22^+0^ to 36^+6^ weeks of gestation. ^＃^AOR **was adjusted for maternal age, parity, and birthplace.** | | | | | | | |

Table S17 presents sensitivity analysis excluded COVID-19-positive pregnant women, enrolling 67,236 deliveries in total. The EPTB rate was markedly higher in the pandemic cohort (0.2%) than the pre-pandemic group (0.1%), with a significant elevated adjusted risk (AOR=2.8, 95%CI 1.7–4.7, P<0.001). By contrast, overall preterm birth rates were similar across groups (7.5% vs. 7.4%, AOR=1.0, P=0.67). Consistent with our main results, the sensitivity test verified that the pandemic only raised the risk of extremely preterm birth, without altering overall preterm birth risk.

Figure S1. Directed acyclic graphs (DAG) for the effects of pandemic period on preterm birth. Solid black lines represent confounding paths that require adjustment (residential status, maternal age, and parity). The red solid line indicates the total causal effect of pandemic period on preterm birth, which is the primary estimate of interest. Dashed black lines represent mediator paths (mode of delivery); adjustment for this variable would block the indirect effect and is therefore excluded from primary models. Mode of delivery is retained only in Supplementary Table S3 as a dedicated sensitivity analysis.
